# Interfacial Passivation Engineering of Perovskite Solar Cells with Fill Factor over 82% and Outstanding Operational Stability on n-i-p Architecture

**DOI:** 10.1021/acsenergylett.1c01811

**Published:** 2021-10-15

**Authors:** Bowen Yang, Jiajia Suo, Francesco Di Giacomo, Selina Olthof, Dmitry Bogachuk, YeonJu Kim, Xiaoxiao Sun, Lukas Wagner, Fan Fu, Shaik M. Zakeeruddin, Andreas Hinsch, Michael Grätzel, Aldo Di Carlo, Anders Hagfeldt

**Affiliations:** † Laboratory of Photomolecular Science, Institute of Chemical Sciences and Engineering, School of Basic Sciences, Ecole Polytechnique Fédérale de Lausanne, CH-1015 Lausanne, Switzerland; ‡ Ångström Laboratory, Department of Chemistry, Uppsala University, Box 523, SE-75120 Uppsala, Sweden; § Centre for Hybrid and Organic Solar Energy (CHOSE), Department of Electronic Engineering, University of Rome Tor Vergata, Rome 00133, Italy; ∥ Institute for Physical Chemistry, University of Cologne, Greinstraße 4-6, 50939 Cologne, Germany; ⊥ Fraunhofer Institute for Solar Energy Systems ISE, Heidenhofstraße 2, 79110 Freiburg, Germany; # Department of Sustainable Systems Engineering (INATECH), Albert-Ludwigs-Universität Freiburg, Emmy-Noether-straße 2, 79110 Freiburg, Germany; ∇ Laboratory for Thin Films and Photovoltaics, Empa-Swiss Federal Laboratories for Materials Science and Technology, 8600 Duebendorf, Switzerland; × Laboratory of Photonics and Interfaces, Institute of Chemical Sciences and Engineering, School of Basic Sciences, Ecole Polytechnique Fédérale de Lausanne, CH-1015 Lausanne, Switzerland; ∞ Institute for Structure of the Matter, National Research Council (ISM-CNR), Rome 00133, Italy

## Abstract

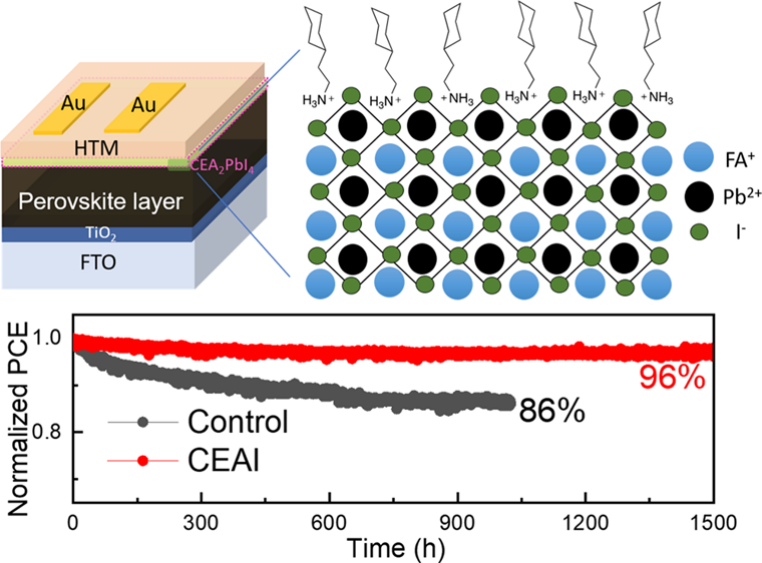

Tremendous efforts have been dedicated toward minimizing the open-circuit voltage deficits on perovskite solar cells (PSCs), and the fill factors are still relatively low. This hinders their further application in large scalable modules. Herein, we employ a newly designed ammonium salt, cyclohexylethylammonium iodide (CEAI), for interfacial engineering between the perovskite and hole-transporting layer (HTL), which enhanced the fill factor to 82.6% and consequent PCE of 23.57% on the target device. This can be associated with a reduction of the trap-assisted recombination rate at the 3D perovskite surface, via formation of a 2D perovskite interlayer. Remarkably, the property of the 2D perovskite interlayer along with the cyclohexylethyl group introduced by CEAI treatment also determines a pronounced enhancement in the surface hydrophobicity, leading to an outstanding stability of over 96% remaining efficiency of the passivated devices under maximum power point tracking with one sun illumination under N_2_ atmosphere at room temperature after 1500 h.

Organic–inorganic hybrid perovskite solar cells (PSCs) are one of the emerging photovoltaic (PV) technologies. Within only the past several years they have achieved photocurrent efficiencies (PCEs) similar to or even higher than those of established PV technologies, from 3.8% in 2009 up to a certified PCE of 25.5% in 2021 in a n-i-p configuration,^1−4^ making it an excellent and promising candidate for the future PV market. However, their inherent properties and the commonly used solution-processing technique result in a significant number of defects at grain boundaries and interfaces to adjacent functional layers. The resulting trap states can lead to undesirable hysteresis, inferior performance, and instability which hinder these remarkable photoabsorber materials from being further scaled up and commercialized in the PV market.^5−8^ Intensive research efforts have been devoted to address these issues, for example, by developing passivation agents to improve the photovoltaic performance and long-term stability.^9,10^ These include organic molecules,^11−13^ polymers,^14,15^ ionic liquids,^16,17^ and ammonium salts.^18−21^ Among these, two-dimensional (2D) perovskites containing larger cations have attracted substantial interest owing to their outstanding stability, especially under moisture conditions, which is important in scaling-up and future commercialization.^22^ Recently, forming thin Ruddlesden–Popper 2D perovskite film on top of the 3D light-absorbing perovskite layer has been developed as an excellent strategy to fabricate highly efficient PSCs.^23−29^ The formation of a 2D layer can effectively passivate the perovskite defects at the interface between the perovskite and hole transporting layers, leading to an improvement in device performance and meanwhile presenting outstanding long-term stability due to their hydrophobicity features. For example, Liu et al. employed F-rich pentafluorophenylethylammonium iodide (FEAI) on the surface of 3D perovskite, achieving perovskite solar cells with over 22% efficiency and outstanding stability in humid conditions.^23^ Kim et al. compared different lengths of alkylammonium iodide, achieving a certified efficiency of 22.9% of the 2D/3D perovskite solar cells with octylammonium iodide (OAI) treatment.^24^ However, most efforts have been dedicated to minimizing the open-circuit voltage deficits and fill factors remain relatively low, especially compared to p-i-n configurations; this limitation stands in the way for their further application in large scalable modules.^30^

Herein, we develop and implement a novel ammonium salt, cyclohexylethylammonium iodide (CEAI), which contains a “chair” conformation cyclohexane and an equatorial substituted ethylammonium group,^31^ as shown in Figure 1. Compared with other well-established passivation salts containing benzene rings, such as PEAI (structure shown in Figure 1), weaker intramolecular interaction of cyclohexanes from CEAI will prevent undesirable aggregation,^19^ leading to a more uniform film and reduced nonradiative recombination, via the formation of a 2D perovskite interlayer between the 3D perovskite and the HTL. As a result, we achieved a target champion device with a PCE of 23.57% (stabilized at 23.4%), *J*_SC_ of 25.04 mA/cm^2^, *V*_OC_ of 1.137 V, and an outstanding FF of 82.6%. Remarkably, the obtained 2D/3D PSCs with CEAI treatment also exhibit excellent long-term stability under operational condition with one sun illumination in N_2_ atmosphere, which retains an impressive 96% of its initial PCE value after aging for 1500 h. In addition, the optimized devices also exhibit superior stability while being exposed to high relative humidity (RH) of 40 ± 20% under dark conditions, retaining 98% of its initial PCE after 600 h of aging.

**Figure 1:**
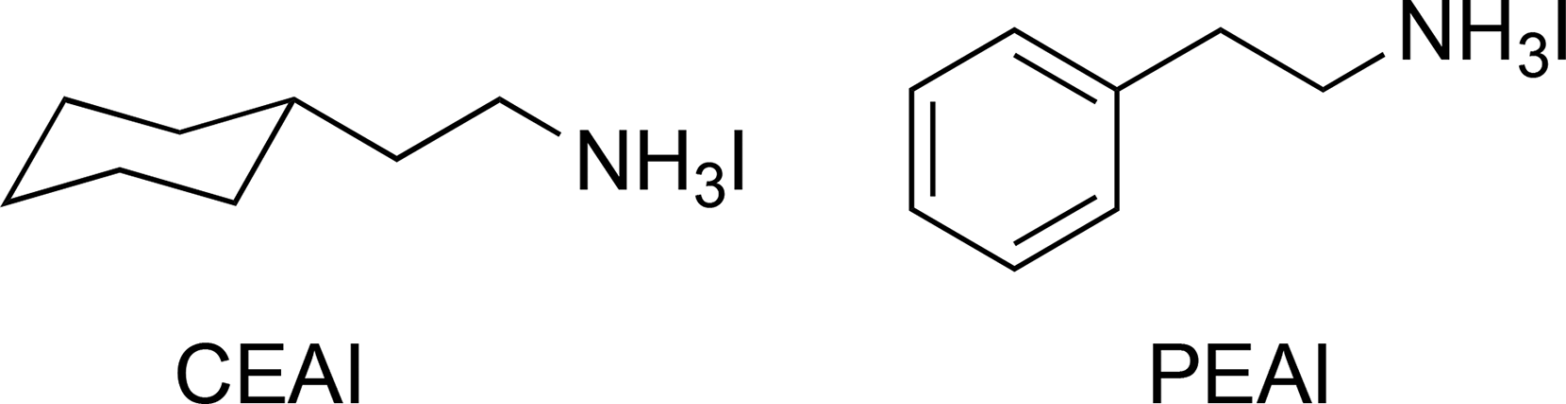
Chemical structures of CEAI and PEAI.

On the basis of our previous work,^20,21^ the mixed-cation and mixed-halide perovskite composition Cs_0.05_MA_0.1_FA_0.85_PbI_2.9_Br_0.1_·0.05PbI_2_ was employed resulting in high quality perovskite films with superior phase stability. The perovskite films were formed by a one-step antisolvent crystallization method and annealed at 100 °C for 1 h. Post-treatment was performed by dropping the solution of CEAI in isopropyl alcohol (IPA) on top of the perovskite film and sequentially annealing it at 100 °C for 10 min.

The surface morphologies of the perovskite films with and without CEAI treatment were observed by high-resolution scanning electron microscopy (HR-SEM) and atomic force microscopy (AFM), as shown in Figure 2. The pristine perovskites in Figure 2a,c display a compact polycrystalline film with some crystals having higher secondary electron current densities (which appear brighter), suggesting the presence of excessive PbI_2_,^32^ which is prone to reside mainly on the grain boundaries and the film surface. However, the additional PbI_2_ disappears after the post-treatment with CEAI and a surface layer formed with a significantly reduced surface roughness, as shown in Figure 2b,d. In comparison with other most commonly used ammonium salts, such as PEAI (Figure S1), the perovskite film treated with CEAI even presents better homogeneity owning to the weaker intermolecular interaction of CEAI. To analyze the composition of the newly formed layer, X-ray diffraction (XRD) measurements were carried out as presented in Figure 2e. In comparison with the pristine perovskite film, new diffraction peaks of the CEAI treated perovskite film appear at 4.7° and 9.5°, which are consistent with the diffraction pattern of the 2D perovskite CEA_2_PbI_4_. Meanwhile, the PbI_2_ peak, shown at 12.6° in the control film, disappears after the post-treatment of CEAI. In addition, by comparison of the XRD pattern with the film of CEAI, it is evidenced that instead of presenting a layer with only CEAI, an additional 2D perovskite layer was formed through a reaction between the remaining PbI_2_ from the pristine perovskite and the newly introduced CEAI. Furthermore, as shown in the grazing-incidence wide-angle X-ray scattering (GIWAXS) measurement in Figure S2, additional Debye–Scherrer rings at low angles for 2D perovskite reveal a preferential crystal orientation with out-of-plane direction.

**Figure 2:**
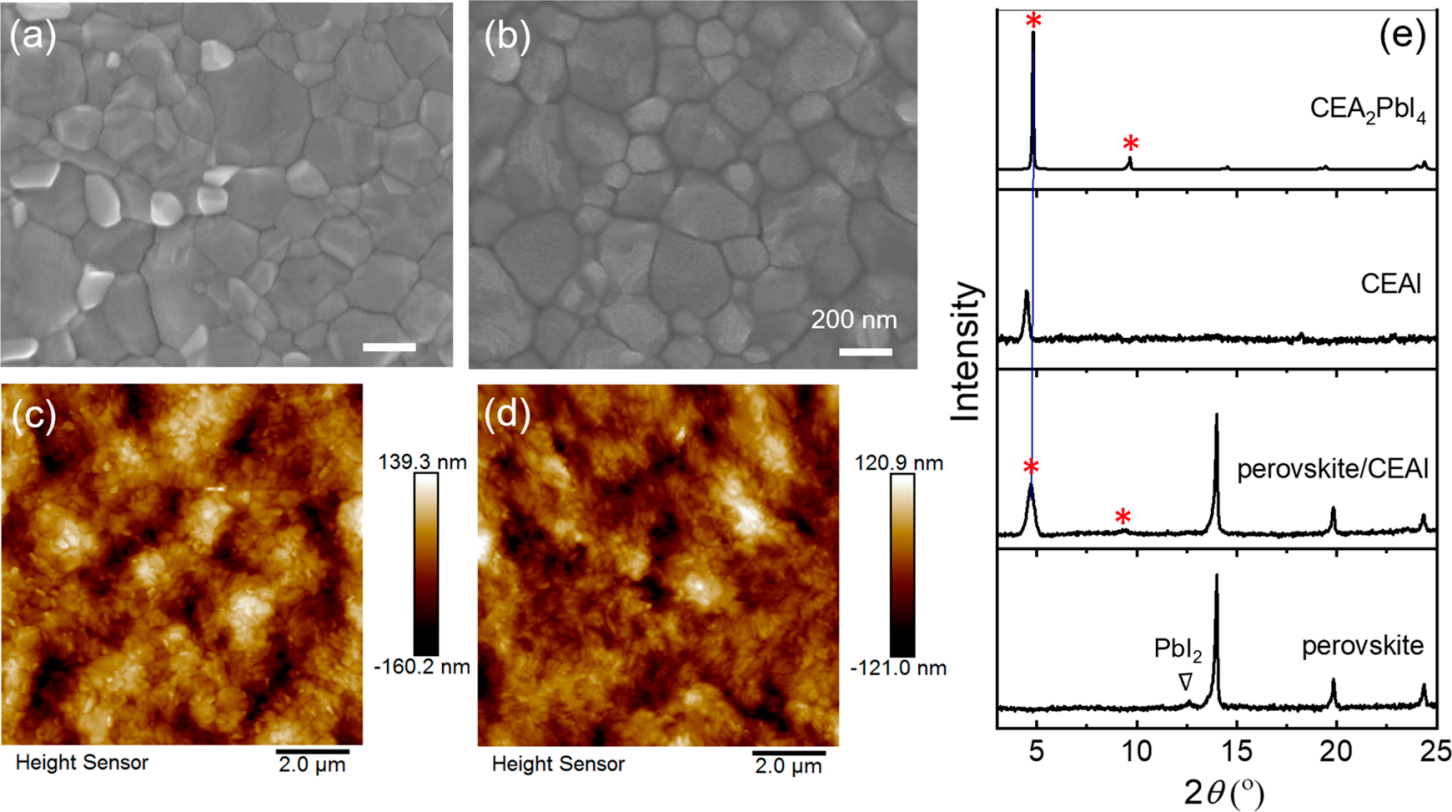
Surface morphology and crystal phases. Top-view SEM images of (a) pristine perovskite and (b) CEAI-treatment film. AFM images of (c) pristine perovskite and (d) CEAI-treated perovskite film. (e) XRD patterns of the pristine perovskite film, CEAI-treated perovskite film, CEAI film, and CEA_2_PbI_4_ film.

Steady-state photoluminescence (PL) and time-resolved photoluminescence (TRPL) decay measurements were carried out to examine the charge recombination behavior of the perovskite film with and without CEAI treatment, as illustrated in Figure S3 and Figure 3a, respectively. A pronounced enhancement in PL intensity is observed upon CEAI treatment. Remarkably, a new feature occurs at around 500 nm wavelength in the PL spectra for the CEAI-treated perovskite film, which is attributed to the crystallization of the very thin 2D layered perovskite on top. This is in agreement with the results obtained by XRD and GIWAXS measurements as discussed above. In addition, the CEAI-treated perovskite film presents a slower photoluminescence decay compared with the pristine perovskite film, as shown in Figure 3a, indicating reduced trap states and significantly suppressed nonradiative recombination upon CEAI treatment.^19,33^

**Figure 3:**
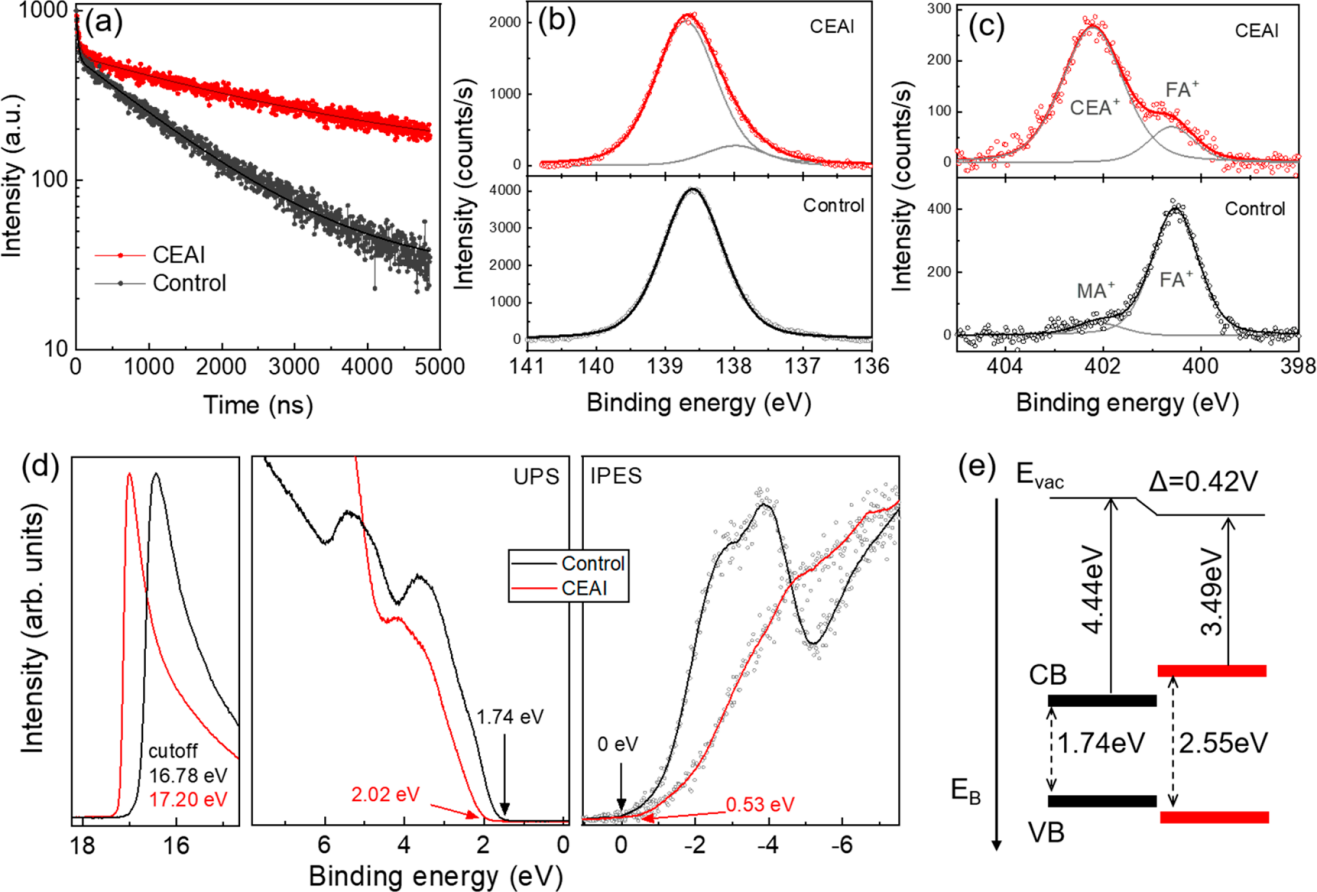
(a) TRPL of the perovskite film with and without CEAI treatment. Photoelectron spectroscopy data: XPS core level signals of (b) the Pb 4f_7/2_ and (c) N 1s of the perovskite film with (red) and without (black) CEAI treatment; in (c) the cation species are indicated from which the core level signals originate. (d) Combined UPS and IPES spectra of the perovskite film with and without CEAI treatment. The arrows indicate the onsets of the VB and CB. (e) Schematic energy level alignment between the perovskite and the 2D capping layer, extracted from the values determined in (d).

X-ray photoelectron spectroscopy (XPS), ultraviolet photoelectron spectroscopy (UPS), and inverse photoelectron spectroscopy (IPES) measurements were carried out to investigate the surface elemental composition, oxidation states, and energy levels of the pristine perovskite film and CEAI-treated perovskite film, as presented in Figure 3. The Pb 4f_7/2_ core level signals of the perovskite films with and without CEAI treatment are shown in Figure 3b. The main peak is located at approximately 138.6 eV in both cases. However, an additional peak at 138.0 eV emerges after the CEAI treatment, which can be associated with the binding Pb in the 2D perovskite surface layer.^34^ The two different organic cation species can be clearly distinguished in the N 1s signal shown in Figure 3c, due to the difference in binding energy. In the pristine perovskite film, the spectrum is dominated by a FA^+^ related peak at 400.5 eV, while the low amount of MA^+^ leads to a weak signal located at 402.1 eV. Both of these values are in good agreement with literature.^35^ Upon treatment with CEAI, a new feature at 402.2 eV dominates the spectrum, which now originates from nitrogen in the CEA^+^ cation. The FA^+^ feature can still be detected; however, it is reduced in intensity by a factor of approximately 6. This suggests that a new FA^+^-deficient layer is formed on the top of the pristine perovskite film due to the introduction of CEAI. By use of UPS and IPES, the relative positions of the valence and conduction bands (VB, CB) of the perovskite film with and without CEAI treatment can be extracted using a linear extrapolation of the density of states at the onsets, as shown in Figure 3d. We find a reduction in work function by 420 meV for the CEAI treated perovskite film, as well as a widening of the bandgap due to the 2D layer formed on top; this likely helps to suppress the charge recombination at the interface between the perovskite and HTL. The resulting schematic energy level diagram is presented in Figure 3e.

To evaluate the influence of CEAI on the photovoltaic performances, PSCs with the n-i-p architecture of FTO/cp-TiO_2_/mp-TiO_2_/3D perovskite/passivation layer/spiro-MeOTAD/Au were fabricated with different CEAI concentrations (10 mM, 20 mM, and 30 mM), as shown in Figure S4. Compared to the control devices without passivation treatment, improvements in all photovoltaic parameters are observed by CEAI treatment. Among those, the devices employing 20 mM CEAI treatment resulted in the best performance, particularly regarding the *V*_OC_ and FF, leading to a significant increase in overall PCE. *J*–*V* curves of the champion devices with and without CEAI treatment are shown in Figure 4a, and the corresponding PV parameters are listed in Table 1. A champion cell with a PCE of 23.57% is achieved, with a *J*_SC_ of 25.04 mA/cm^2^, *V*_OC_ of 1.137 V, and a remarkable FF of 82.6% in the reverse scan direction. The stabilized power output was carried out by maximum power point tracking (MPPT) for 5 min, and the final stabilized PCE of the champion device is 23.4%, shown in Figure 4b. The photocurrent density (*J*_SC_) shows a comparable value of around 25 mA/cm^2^ and the corresponding incident photon-to-current efficiency (IPCE) is presented in Figure S5, where the integrated *J*_SC_ is well aligned with *J*_SC_ from *J*–*V* curves. In addition, negligible hysteresis is observed between the forward and reverse scans of the CEAI treated device, whereas the control device suffers from severe hysteresis. Moreover, the ideality factor (*n*) was measured to examine the effect of CEAI treatment on the Shockley–Read–Hall recombination of the device, as shown in Figure 4c. It is observed that the slope of *V*_OC_ versus the natural logarithm of light intensity for PSC treated with CEAI (1.22 *k*_B_*T*/*q*) is smaller than that of the control device without passivation treatment (1.78 *k*_B_*T*/*q*), indicating a suppressed trap-assisted charge recombination through CEAI treatment.^36,37^ The reduction of nonradiative recombination can also be assessed by studying the electroluminescence quantum efficiency (EL_EQE_) of CEAI treated devices compared to the control. As shown in Figure S6, the EL_EQE_ increases from 0.3% to 8%, in accordance with the large increase of *V*_OC_.^38^ Similarly, a transient photovoltage measurement allowed study of the increase of the charge lifetime in a device, demonstrating the longer lifetime in CEAI treated devices, shown in Figure S7. It is worth noting that the devices passivated with CEAI show more improvement in device performance compared to those passivated with PEAI, as illustrated in Figure S4.

**Figure 4:**
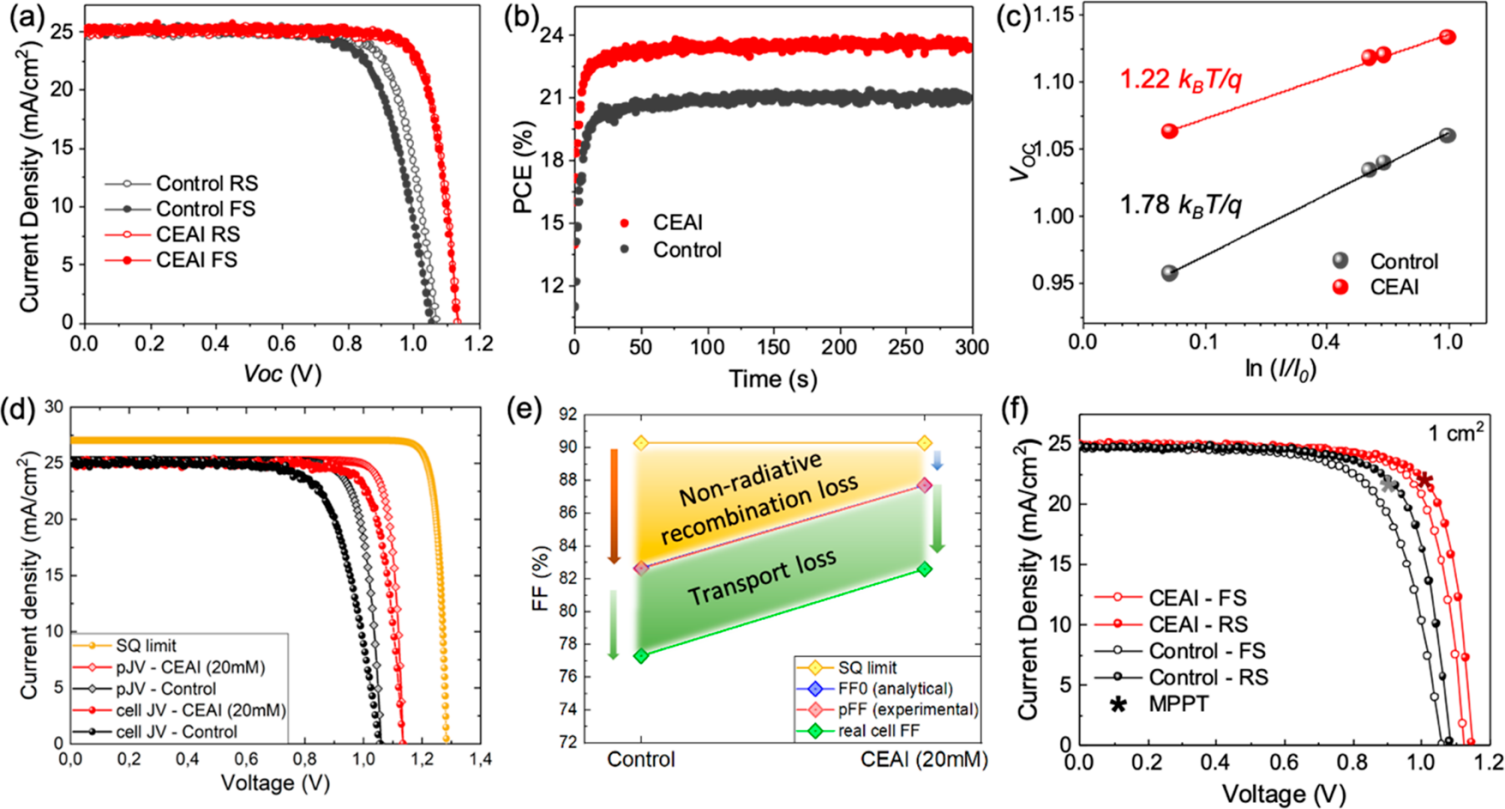
(a) *J*–*V* curves of the control (black) and CEAI-treated (red) devices, where reverse scan (RS) and forward scan (FS) are indicated as open symbols and solid symbols, respectively. (b) Power output of the device employing CEAI treatment and the control devices at maximum power point as a function of time. (c) *V*_OC_ versus logarithm of light intensity of the PSCs with and without CEAI treatment devices. (d) *J*–*V* curves of an ideal device with a bandgap of 1.56 eV limited only by the radiative recombination (Shockley–Quisser limit), pseudo-*JV* curves (constructed from the light-intensity-dependent *V*_OC_ measurement) of cells with and without CEAI passivation, and their actual *J*–*V* curves (the area in between represents transport loss). (e) FF in the case of radiative limit for devices with this bandgap, theoretical FF (FF_0_) calculated from eq 1 and experimentally obtained pseudo-FF, both of which demonstrate the FF in the absence of charge transport losses, as well as the FF of the actual devices. (f) *J*–*V* curves of the champion 1 cm^2^ devices measured with the four-wire split method. The star indicates the *J*_MPP_ and the *V*_MPP_ after 60 s of MPPT.

**Table 1:** Champion Photovoltaic Parameters of PSCs with and without CEAI Treatment

	*J*_sc_ (mA/cm^2^)	*V*_OC_ (V)	FF (%)	PCE (%)	HI^c^(%)
control-RS^a^	25.12	1.074	77.8	20.99	6.5
control-FS^b^	25.09	1.065	73.4	19.61	
CEAI-RS	25.04	1.137	82.6	23.57	1.5
CEAI-FS	24.97	1.133	82.1	23.21	

a Reverse scan.

b Forward scan.

c HI = (PCE_RS_ – PCE_FS_)/PCE_RS_.

The above results indicate that CEAI treatment can effectively prolong charge carrier lifetime and reduce the nonradiative recombination rate, which results in the expected improvements not only of *V*_OC_ and diode ideality factor (*n*) but also of FF. Therefore, to gain more insight into the origins of FF improvement by CEAI treatment, we differentiate between the FF losses caused by the nonradiative recombination and by charge transport. To do that, we calculate the theoretical FF in the absence of charge-transport losses (FF_0_) coupled with light-intensity-dependent measurement of *J*_SC_ and *V*_OC_ values to construct a pseudo-*JV* curve as shown in Figure 4d, which represents a *J*–*V* curve without charge transport losses. More detailed discussion on the calculation of FF_0_ and pseudo *J*–*V* curve can be found in the Supplementary Note 1. The obtained pseudo-FF (pFF) of control cells and cells with 20 mM CEAI treatment are 82.6% and 87.7%, respectively, which correlate remarkably well with the calculated FF_0_, which was determined to be 82.7% and 87.7% for control and CEAI-treated devices, respectively.

Comparing these values with the theoretical maximum fill factor FF_max_ obtained from the Shockley–Queisser approximation (more details can be found in Supplementary Note 2), it can be seen that devices with the optimal CEAI-treatment show exceptionally low FF losses due to nonradiative recombination of <3%_abs_, while in control devices the nonradiative recombination loss is close to 8%_abs_ (Figure 4e). We note that the difference between the pFF (or FF_0_) and the current–voltage FF is nearly the same for both device configurations with 5.1%_abs_ for CEAI-treated and 5.3%_abs_ for control devices. This clearly indicates that CEAI treatment does not cause any additional charge transport hindrance while effectively passivating the nonradiative recombination sites. We highlight that in the case of control cells, nonradiative recombination is in fact the major FF loss, which can be strongly reduced by interfacial treatment with CEAI. Hence, we attribute the increase in FF in the passivated cells entirely to the reduction of trap-assisted recombination rate at 3D perovskite surface, via formation of low-dimensional CEA_2_PbI_4_ 2D perovskite as mentioned above.

To further assess the quality of CEAI-treated cells, we fabricated devices with active areas of 1 cm^2^, with summarized PV parameters in Table S1. The CEAI treated devices have obtained a PCE of 20.4%, while control devices resulted in an efficiency of 16.3% by using the standard measurement with two-wire method, which is depicted in the scheme shown in Figure S8. However, it is known that the fabrication of highly efficient large area cells requires uniform and shunt-free layers over the active area and the minimization of the resistive losses in the less conductive electrode (i.e., the transparent conductive oxide) to avoid a reduction of the FF. While the former requirement is mainly related to the fabrication protocol, the latter is strongly influenced by the design of the cell and by the method used for contacting and measuring the device. We can break down the resistive losses among the ones in the active area (intrinsic losses) and the ones in the region that connect the active area to the measurement system (extrinsic losses). Intrinsic losses are a constituent part of the device and will impact the performance of large area devices such as modules and panels. For example, increasing the cell width of a module from 3 to 10 mm will cause a 5% reduction of FF, and this loss cannot be avoided for large cells.^39^ On the other hand, extrinsic losses are particular for this configuration and are not present in optimized modules: the design of modules is aimed to reduce the ohmic losses by a series connection of narrow cells. For this reason, the extrinsic losses should not be taken in consideration when evaluating large area cells as an intermediate step toward modules. These losses can be significant since highly efficient PSC of 1 cm^2^ can have a large *I*_MPP_ of about 23 mA, meaning that a 5 Ω resistance will cause a drop of 115 mV in the *V*_MPP_. However, it is possible, as proved in this study, to eliminate ohmic losses by using different configurations of a four-wire measurement, which acting on the measurement setup allows the evaluation of highly efficient 1 cm^2^ cells without the need for the optimization of the cell layout. A scheme of 1 cm^2^ devices and the contacting schemes for the measurements are shown in Figure S8. The minimization of the resistive losses can be obtained by placing the two negative contacts on two different sides of the active area (either on the opposite sides or on adjacent sides), as illustrated in Figure S9. As expected, the change of the position of the contact is only affecting the FF while it is not changing the *V*_OC_ or the *J*_SC_. It is possible to achive large improvement in FF by using the four-wire split measurement. With this measurement, a large area CEAI-treated cell displayed a PCE_MPPT_ of 22.4% and remarkable FF of 78.2%. Meanwhile, as shown in Figure 4f and Table S1, the cell still retains a high *V*_OC_ of 1.149 V and a *J*_SC_ of 24.9 mA/cm^2^. Even if there is a small hysteresis in the *JV* curve, the MPPT confirmed the results obtained with the reverse scan (see Figure S10). The CEAI treatemt showed the same effect on 1 cm^2^ cells, by increasing the *V*_OC_, the FF and reducing the hysteresis. Another advantage of this method is the possibility of using the *JV* curves of 1 cm^2^ as input for simulating the efficiency of a module. As an example (see Figure S11 for additional parameters), a CEAI treated module could achieve an efficiency on aperture area of over 21%.

Finally, long-term stability of the devices with and without CEAI treatment was monitored under maximum power point tracking in nitrogen atmosphere at room temperature under constant full sun illumination for more than 1000 h. As shown in Figure 5a, the unencapsulated control device exhibits already 14% loss in normalized PCE after 1000 h aging under operational conditions, whereas the device passivated with CEAI remains at an impressive stability of 96% from its initial PCE value after 1500 h MPPT. In addition, the property of the newly formed 2D perovskite along with the cyclohexylethyl group introduced by CEAI treatment determines a pronounced enhancement in the surface hydrophobicity, compared to the bare perovskite. Accordingly, the contact angle measurements of the perovskite film with and without CEAI treatment are shown in Figure 5b (inset), where the CEAI treated film shows excellent hydrophobicity. Consequently, the shelf life stability in humid environment was investigated. The device performance was monitored while exposing them in ambient condition with relative humidity (RH) of 40 ± 20% under dark conditions. As shown in Figure 5b, after over 600 h of aging, the PCE of the control device suffers a rapid decay of over 20% from its initial value, whereas, in sharp contrast, the target devices with CEAI treatment exhibits outstanding long-term stability under the same conditions, which still retains about 98% PCE of its initial value.

**Figure 5:**
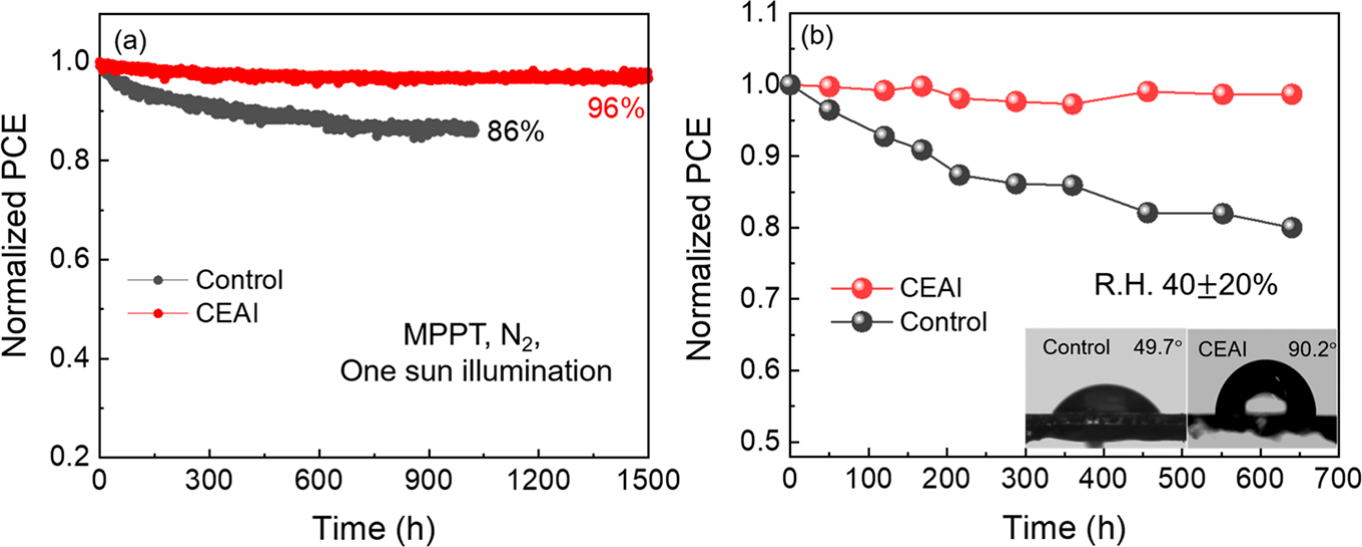
Long-term stability of the unencapsulated control device (black) and the optimized device with CEAI treatment (red) under (a) MPPT in N_2_ atmosphere with one sun illumination at 25 °C; (b) storage conditions with RH of 40 ± 20% in dark at room temperature, where inset images are the contact angle of water droplet on perovskite film with and without CEAI treatment.

In summary, efficient and operational stable PSCs have been demonstrated by interfacial engineering with CEAI treatment. We attribute the simultaneous enhancement of *V*_OC_ and FF to the reduction of nonradiative recombination losses at the 3D perovskite surface, via the formation of a 2D perovskite interlayer, which also determines the outstanding stability of the devices under various aging conditions. The passivated cells exhibited a long operational stability, by retaining 96% of the starting efficiency after 1500 h of maximum power point tracking at full sun illumination. More interestingly, by comparing with the standard two-wire method, we proposed a four-wire split method for *IV* measurements, which has been proved to be able to largely minimize the intrinsic series resistance losses of a solar cell. Such measurement setup allows the evaluation of highly efficient 1 cm^2^ cells without the need for the optimization of the cell layout and can ease the evaluation of large area cells as an intermediate step for fabricating highly efficient modules.

## Supporting Information Available

Synthesis of CEAI, PSCs devices fabrication, and characterization, top-view SEM image of PEAI treated perovskite film, GIWAXS images, steady-state PL, statistical box charts of device photovoltaic parameters, IPCE and integrated photocurrent density, EL spectrum, TPV decay time, champion photovoltaic parameters of 1 cm^2^ PSCs, schemes of two-wire and four-wire configuration, *J*–*V* curevs of 1 cm^2^ PSCs, MPPT of 1 cm^2^ PSCs, and simulated maximum efficiency by four-wire split measurement (PDF)
