# Supplementary material for: Interfacial Passivation Engineering of Perovskite Solar Cells with Fill Factor over 82% and Outstanding Operational Stability on n-i-p Architecture
Source: ACS Energy Lett. 2021 Oct 15;6(11):3916–23. doi: 10.1021/acsenergylett.1c01811 (PMC8593894; doi:10.1021/acsenergylett.1c01811)
Supplement: Supplementary file 1 — nz1c01811_si_001.pdf [file nz1c01811_si_001.pdf]

Interfacial passivation engineering of perovskite solar cells with fill factor over 82% and outstanding operational stability on n-i-p architecture

Bowen Yang,[a,b†*] Jiajia Suo,[a,b†] Francesco Di Giacomo,[c*] Selina Olthof,[d] Dmitry Bogachuk,[e,f] YeonJu Kim,[a] Xiaoxiao Sun,[g] Lukas Wagner,[e,f] Fan Fu,[g] Shaik M. Zakeeruddin,[h] Andreas Hinsch,[e] Michael Grätzel,[h] Aldo Di Carlo,[c,i] Anders Hagfeldt[a,b]*

[a]Laboratory of Photomolecular Science, Institute of Chemical Sciences and Engineering, School of Basic Sciences, Ecole Polytechnique Fédérale de Lausanne, CH-1015 Lausanne, Switzerland

[b]Department of Chemistry - Ångström Laboratory, Uppsala University, Box 523, SE-75120 Uppsala, Sweden

[c] Centre for Hybrid and Organic Solar Energy (CHOSE), Department of Electronic Engineering, University of Rome Tor Vergata, Rome 00133, Italy

[d]University of Cologne, Institute for Physical Chemistry, Greinstrasse 4-6, 50939 Cologne, Germany

[e]Fraunhofer Institute for Solar Energy Systems ISE, Heidenhofstr. 2, 79110 Freiburg, Germany

[f]Department of Sustainable Systems Engineering (INATECH), Albert-Ludwigs-Universität Freiburg, Emmy-Noether-str. 2, 79110 Freiburg, Germany

[g]Laboratory for Thin Films and Photovoltaics, Empa−Swiss Federal Laboratories for Materials Science and Technology, 8600 Duebendorf, Switzerland

[h]Laboratory of Photonics and Interfaces, Institute of Chemical Sciences and Engineering, School of Basic Sciences, Ecole Polytechnique Fédérale de Lausanne, CH-1015 Lausanne, Switzerland

[i]Institute for Structure of the Matter, National Research Council (ISM-CNR), Rome 00133, Italy

†Both authors contributed equally to this work.

*Corresponding authors

B.Y.: bowen.yang@kemi.uu.se, F. D. G.: francesco.di.giacomo@uniroma2.it, A. H.: anders.hagfeldt@uu.se

Experimental Section

Synthesis of CEAI

2-Cyclohexylethylamine (0.63 g, 5.0 mmol) (TCI) was dissolved in 20 mL MeOH at 0 ^{o}C, hydroiodic acid solution (1 mL) (Sigma-Aldrich, 57% in water) was added dropwise. After the mixture was stirred for 1 h, the solution was removed to room temperature and stirred for another 2 h. After solvent evaporation, the resulted solid residue was washed with diethyl ether three times, affording white solid product (1.10 g, 87 % yield). ^{1}H NMR (400 MHz, DMSO) δ 7.56 (s, 3H), 2.87 – 2.72 (m, 2H), 1.73 – 1.56 (m, 5H), 1.50 – 1.35 (m, 2H), 1.35 – 1.05 (m, 4H), 0.96 – 0.78 (m, 2H); ^{13}C NMR (101 MHz, DMSO) δ 36.87, 34.45, 34.19, 32.38, 25.89, 25.56.

Substrate

Fluorine doped tin oxide (FTO) substrates (NSG-10) were chemically etched by zinc powder and 4 M HCl solution and sonicated in 2% Hellmanex water solution for 30 min, acetone for 15 min and ethanol for 15 min, respectively. Then, all substrates were further cleaned by UV-Ozone for 15 min. Then, a compact TiO_2 layer was deposited on cleaned FTO substrates via spray pyrolysis deposition from a precursor solution of titanium diisopropoxide bis(acetylacetonate) (Sigma-Aldrich) in anhydrous ethanol (Acros), with oxygen as carrier gas. Substrates were heated at 450 ^{o}C and kept at this temperature for 15 min before and 30 min after the spray of the precursor solution, then left to cool down to room temperature. Mesoporous TiO_2 layer was spin-coated at 4000 rpm for 20 s, with the acceleration rate of 2000 rpm/s, using a 30 nm TiO_2 paste (Dyesol 30 NR-D) diluted in ethanol with 1:6 volume ratio. After the spin-coating, the substrates were dried at 80 ℃ for 10 min and then sintered at 450 ^{o}C for 30 min under dry air flow.

Perovskite layer

The perovskite precursor solution was prepared by dissolving a mixture of cesium iodide (0.07 mmol, TCI Co. Ltd.), methylammonium bromide (0.14 mmol, Dyenamo), formamidinium iodide (1.19 mmol, Dyenamo), lead iodide (1.45 mmol, Alfa Co. Ltd.) in 1 mL mixture of DMF and DMSO (DMF:DMSO=4:1 v/v, Acros). The perovskite solution was spin-coated through two-step program (1000 rpm for 10 s and 6000 rpm for 20 s) with pouring chlorobenzene as an anti-solvent 5s before the end of the second step. Then the substrates were annealed at 100 ^{o}C for 1 h in dry air. The CEAI was dissolved in IPA and the solution was spin-coated at 4000 rpm for 20s on the as-prepared perovskite films and dried on a hot plate at 100 ^{o}C for 10 min.

Hole transporting layer and Au top contact

The substrates were cooled down to room temperature after annealing the perovskite. A spirofluorene-linked methoxy triphenylamines (spiro-MeOTAD, Merck) solution was deposited by spin coating at 4000 rpm for 20 s, as hole-transporting material. 90 mg spiro-MeOTAD was dissolved in 1 ml chlorobenzene, doped by 20.6 µL bis(trifluoromethylsulfonyl)imide lithium salt solution (520 mg/mL LiTFSI in acetonitrile), and 35.5 µL 4-*tert*-butylpyridine (tBP, Sigma-Aldrich). Finally, 80 nm of Au top electrode was deposited through thermal evaporator under high vacuum with an active area of 0.16 cm^2.

Characterization

The solar cell devices were measured using a 300 W Xenon light source (Oriel). The spectralmismatch between AM 1.5 G and the solar simulator was calibrated by a Schott K113 Tempax filter (Prazosopms Gas & Optik GmbH). The light intensity was calibrated with a silicon photodiode with an IR-cutoff filter (KG2, Schott). Current-voltage characteristics were applied by an external voltage bias while measuring the corresponding current with Keithley 2400 under ambient air condition. The voltage scan rate was 50 mV/s. The devices were covered with a black metal mask with an active area of 0.16 cm^2. Incident photon to current efficiency (IPCE) was carried by a commercial apparatus (Aekeo-Ariadne, Cicci Research s.r.l.). The top-view and cross-section morphologies of the samples was characterized using a high-resolution scanning electron microscope (Zeiss Merlin) with an in-lens secondary electron detector. The X-ray diffraction patterns were recorded with PANalytical Empyrean system with a PIXcel-1D detector, Bragg-Brentano beam optics and parallel beam optics. Light source is from copper Kα beam filtered with nickel β filter. Diffraction spectra were characterized between 2-theta of 10° and 70° at a scan rate of 1° per minute with the step width of 0.02^o. Steady state photoluminescence (PL) spectra were obtained with an Andor Kymera 193i spectrometer equipped with a silicon CCD camera (iDus DU240A-OE). Excitation was provided by a CW laser (Obis, 422 nm, 1 mW, beam diameter at 1/e2 0.9±0.1 mm) for PL. ^{1}H NMR and ^{13}C NMR measurements were performed on Bruker AvanceIII-400 MHz NMR spectrometer. The operational stability of the devices was measured under a white light-emitting diode lamp with biologic MPG2 potentiostat under N_2 gas flow at maximum power point tracking (MPPT). GIWAXS were measured on a Bruker D8 Discover Plus instrument equipped with a rotating anode and a Dectris Eiger2 500K detector. The primary beam path was collimated by a 1.0 mm micro-mask (after a focussing Göbel mirror), followed by a 0.5 mm micromask, followed by a 0.3 mm double-pinhole collimator. The detector was placed at 178 mm from the sample and 2D images were acquired for 300 seconds at 2° Theta (incidence

angle). 2D images were integrated using EVA. Photoelectron spectroscopy measurements were performed in a custom built ultra-high vacuum system. For UPS, a monochromatic He excitation source (VUV5k Scienta) was used with a photon energy of 21.22 eV. The samples were biased at -8 V and a pass energy of 2 eV was used. For XPS measurements the excitation energy was 1253.6 eV, generated by a non-monochromatic Mg K_{α} source (VG). The pass energy was 10 eV and the samples were grounded during the measurement. For UPS and XPS the kinetic energy of the generated photoelectrons were measured using a hemispherical energy analyzer (Phoibos 100, Specs). For IPES, a low energy electron gun (ELG-1, Kimball) was employed to create electrons with kinetic energies ranging from 5 to 16 eV while the generated photons were detected using a bandpass photon detector (SrF2/ NaCl bandpass, IPES 2000, Omnivac); the sample current was set to 2 μA. The AFM characterization were carried out using an AFM microscope (Bruker ICON3) in air. A silicon nitride tip (scanasyst-air) with a radius of 10 nm was used as the probe. The cantilevers' spring constant and resonant frequency are 0.4 N/m and 70 kHz, respectively. Electroluminescence measurement were carried out with the Arkeo platform (Cicci Research), using a FDS1010 photodiode. The same tool was used to measure the EL spectrum and to carry out the transient photovoltage measurement. The 4-wire measurement were carried out with a Keithely 2420 and a customized LabView application.

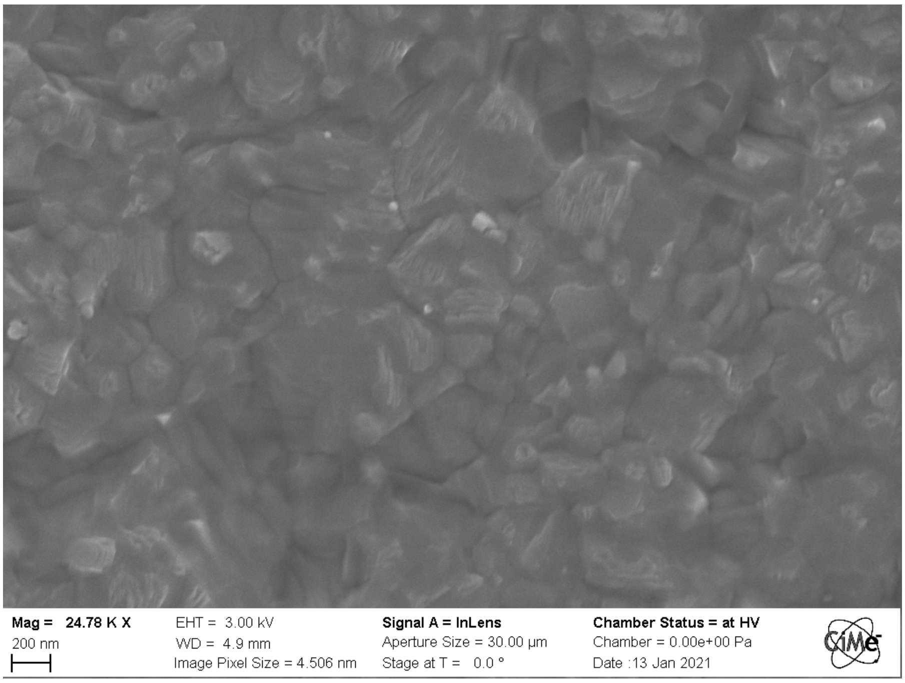

Figure S1. Top-view SEM image of PEAI-treated film.

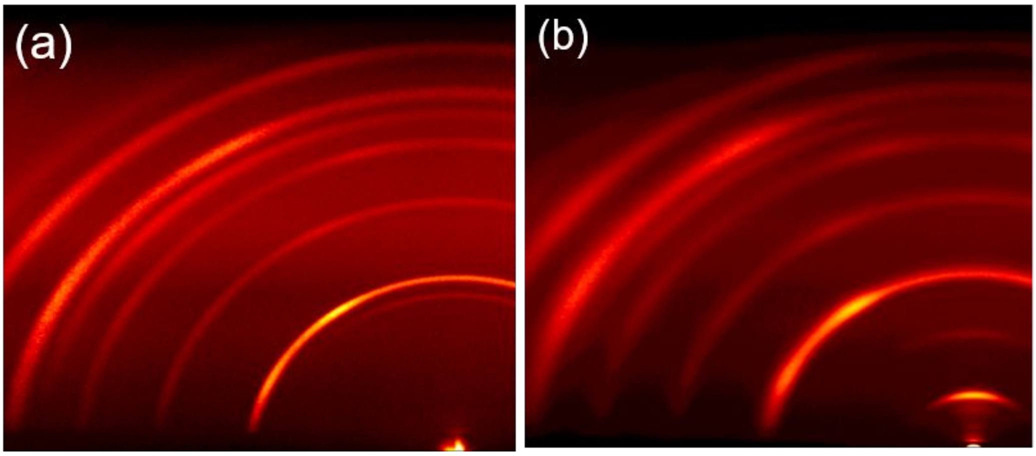

Figure S2. GIWAXS images of the (a) pristine perovskite and (b) CEAI-treatment film.

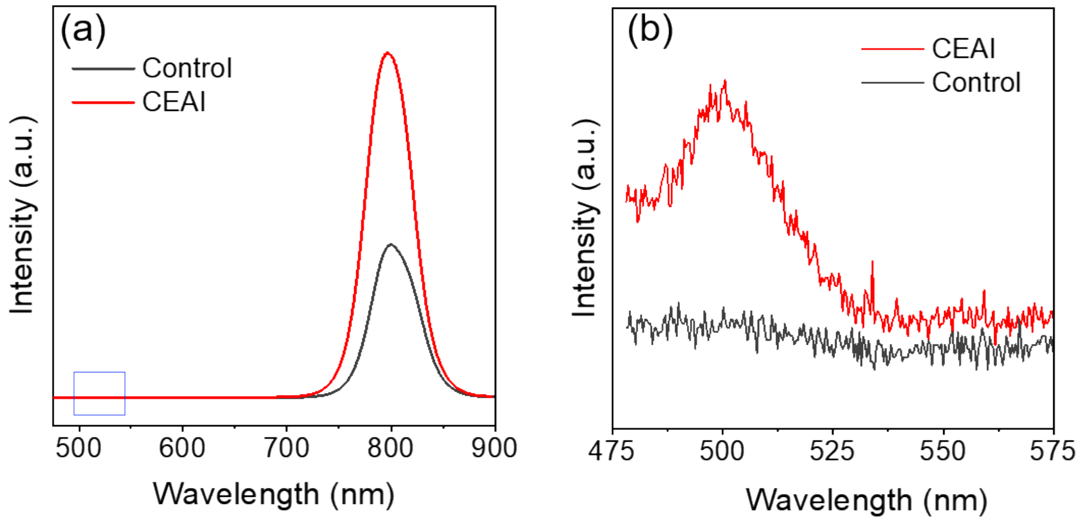

Figure S3. Steady-state PL of perovskite film with and without CEAI treatment.

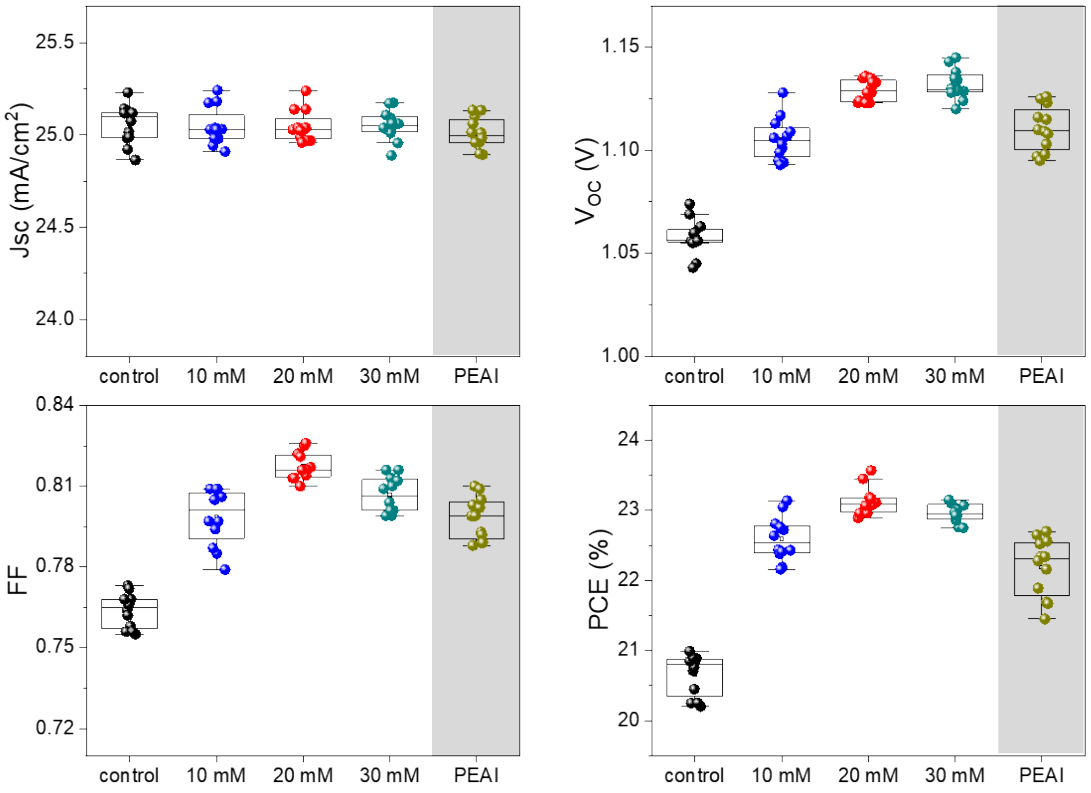

Figure S4. Statistical box charts for the photovoltaic parameters of (a) J_{SC}, (b) V_{OC}, (c) FF, and (d) PCE of the 12 devices each condition without and with CEAI treatment of different concentrations (10 mM, 20 mM and 30 mM), PEAI (20 mM) treatment.

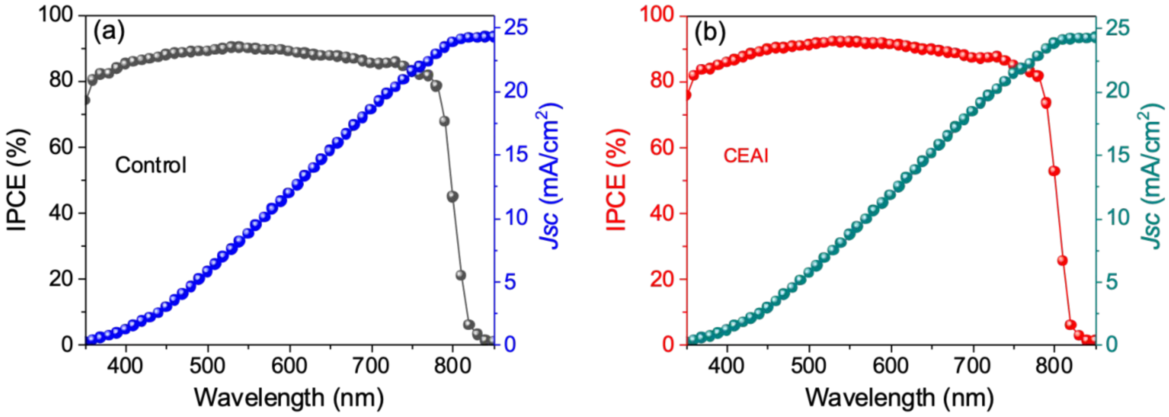

Figure S5. IPCE and integrated photocurrent density of devices with and without CEAI treatment.

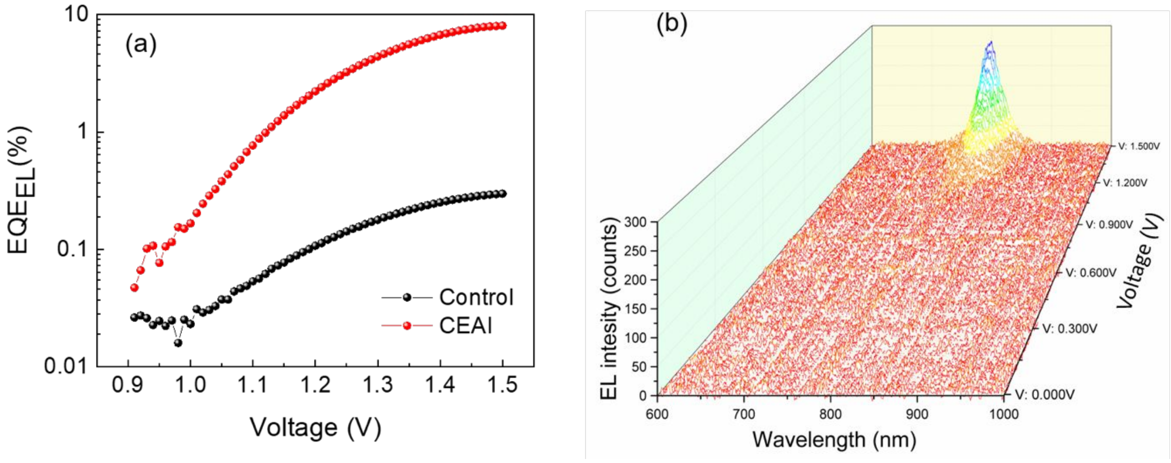

Figure S6. (a) Comparison of the electroluminescence quantum efficiency for a CEAI-treated cell compared to a control cell. (b) EL spectrum of the CEAI treated device at different voltages.

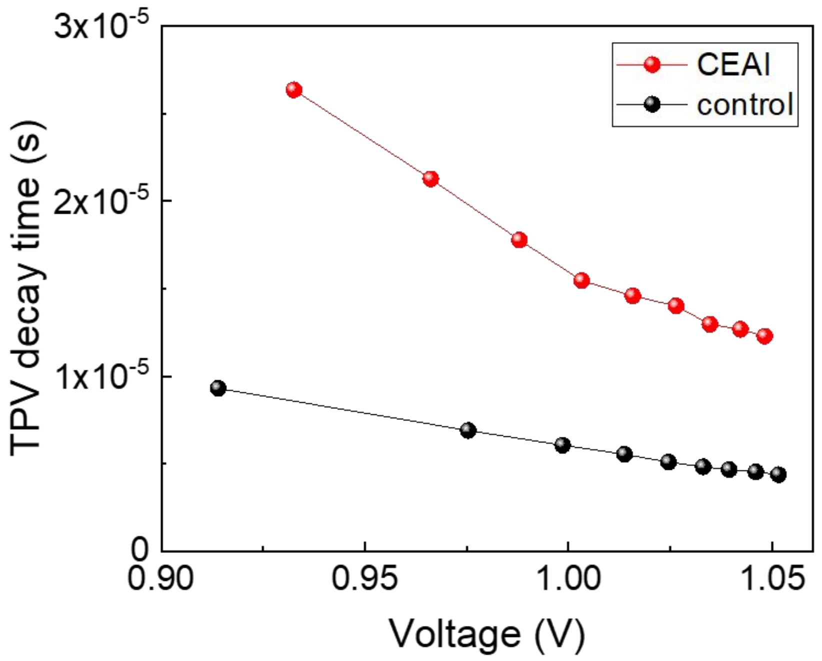

Figure S7. Comparison of the decay time of transient photovoltage measurement for a CEAI-treated cell compared to a control cell.

Supplementary Note 1

The theoretical FF in the absence of charge-transport losses (FF_0) was calculated via the analytical expression proposed by Green:[1]

$$FF_0 = \frac{v_{oc} - \ln(v_{oc} + 0.72)}{v_{oc} + 1} \qquad \textbf{Eq. 1}$$

where $v_{oc} = \frac{qV_{oc}}{nk_BT}$, q denotes elementary charge, V_{oc} – open-circuit voltage, n – ideality factor, k_B – Boltzmann constant and T – temperature. This empirical approximation has regularly proven to correlate well with the experimentally obtained values for silicon solar cells.[2]

From the obtained light-intensity-dependent measurement of V_{OC} and extrapolated J_{SC} values, pairs of J_{SC}-V_{OC} at different light intensities have been obtained. Since the measurement is conducted under the open-circuit condition, no transport losses are present in this case because no current is drawn from the cell.

Pseudo current density (pJ) is obtained by:

$$pJ(I) = J_{SC@1Sun} - J_{SC}(I)$$

where each specific light intensity value (I) corresponds to a certain J_{SC}-V_{OC} pair. For a more accurate determination of the pseudo-maximum-power-point (and thus pFF), measured V_{OC}-J_{SC} pairs were fitted to the V_{OC} expression shown below, based on the measured ideality factor and $J_{sc@1Sun}$.

$$V_{oc}(I) = \frac{nk_BT}{q}\ln\left(\frac{J_{ph}(I)}{J_0\,(I)} + 1\right)$$

where J_{ph} is the photogeneration current (approximated by J_{SC}) and J_0 is the dark saturation current.

Plotting the subtracted J_{SC} at each light intensity from the J_{SC} at one sun illumination (i.e. plotting pJ) against V_{OC} gives pseudo J-V curve, which is essentially a light J-V curve without series resistance. However, the non-radiative recombination processes are still present in the cell, even though no current is drawn from it. Thus, pFF obtained from the pJV curve represents a case where non-radiative recombination losses of the FF are still present, but transport losses (e.g. transport of carriers through perovskite bulk, their extraction and transport through the

charge-selective layers, losses at interfaces) are not. This gives a unique opportunity to disentangle between the contributions of transport and non-radiative recombination losses in a cell.

Supplementary Note 2

To get the FF value in the absence of transport and non-radiative losses – FF_{max}, Shockley-Queisser approximation can be used, which considers that the only loss of the photo-excited charge carriers happens due to radiative recombination process:

$$J(V) = J_{ph} - J_0 e^{\frac{qV}{nk_B T}}$$

Since J_{ph} and J_0 depend on the semiconductor absorption spectrum, its bandgap energy must be known to calculate FF_{max}. Based on the PL spectra shown in **Figure S3**, the band gap of the studied perovskite was determined to be 1.56 eV. Thus, a *J-V* curve in the case of Shockley-Queisser limit has been calculated via publicly available python-based script (https://github.com/marcus-cmc/Shockley-Queisser-limit) and plotted in **Figure 4d** (yellow *J-V* curve), having a FF_{max} of 90.3% for a perovskite absorber investigated in this study.

Table S1. Champion photovoltaic parameters of 1 cm^2 PSCs with CEAI treatment

	Measurement	*Jsc* (mA/cm^2)	V_{OC} (V)	FF (%)	PCE (%)	PCE_{MPPT} (%)
CEAI-RS	2-wire	25.1	1.148	70.8	20.4	/
Control-RS	2-wire	24.9	1.055	62.1	16.3	
CEAI-RS	4-wire split	24.9	1.149	78.2	22.37	22.4
CEAI-FS	4-wire split	25.1	1.137	75.6	21.54	
Control-RS	4-wire split	24.6	1.084	74.8	19.98	19.3
Control-FS	4-wire split	24.8	1.074	68.4	18.21	

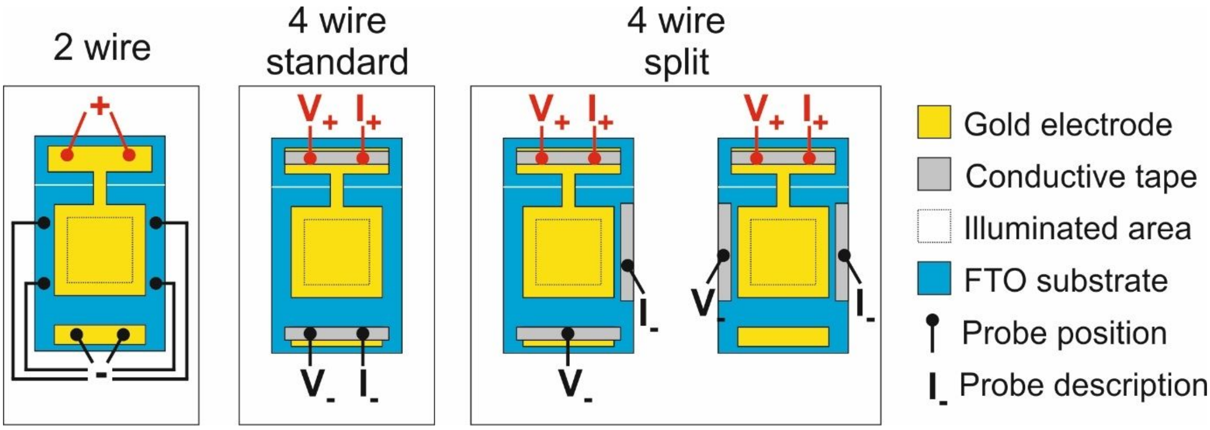

Figure S8. Schemes of the connection used to measure the 1 cm^2 devices. In the 2-wire configuration, the V_+ / I_+ and V_- / I_- are shunted before the connection to the contact probes. The two configurations of the 4-wire split have been showing the same results.

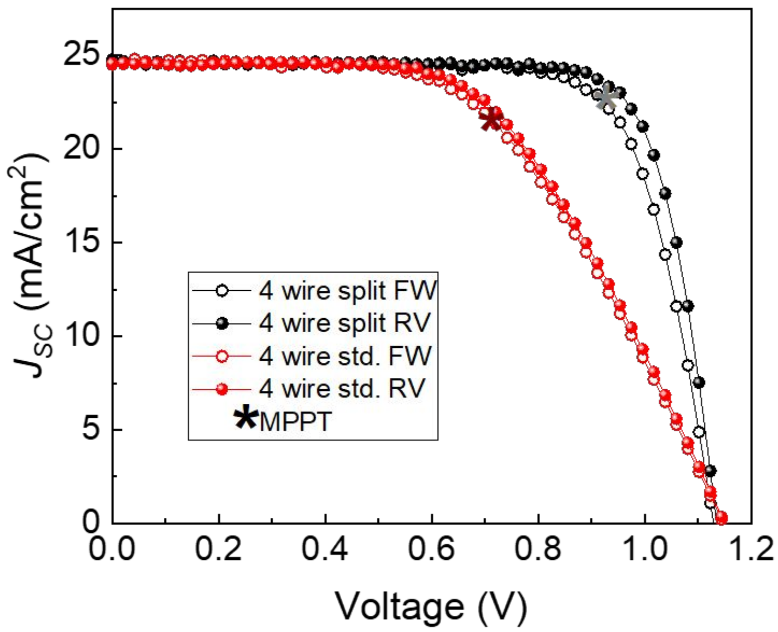

Figure S9. Effect of the different 4-wire connection schemes on the JV curves of a 1 cm^2 cell. The star indicates the value of MPPT after a minute of tracking.

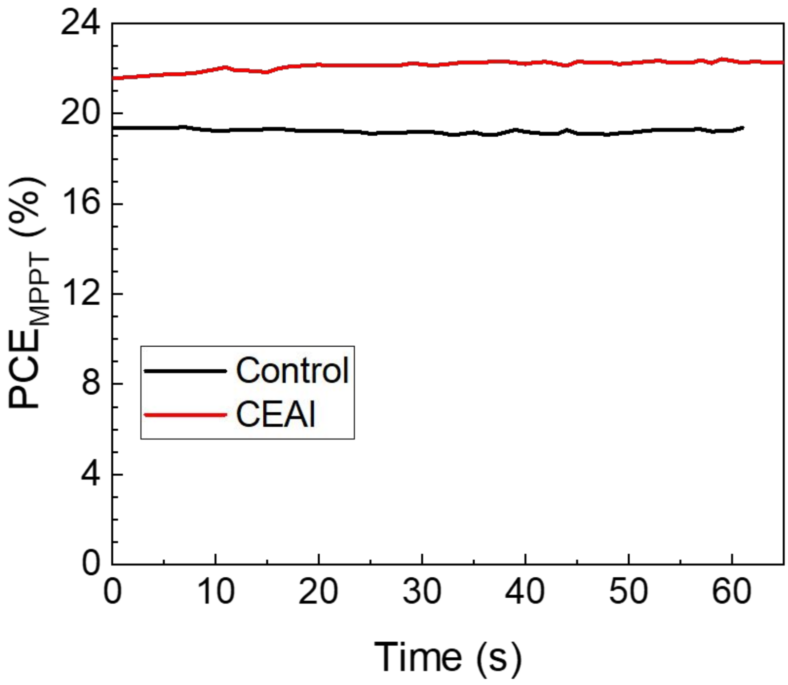

Figure S10. Maximum power point tracking (4-wire split method) of the champion 1 cm^2 cells presented in Figure 4f.

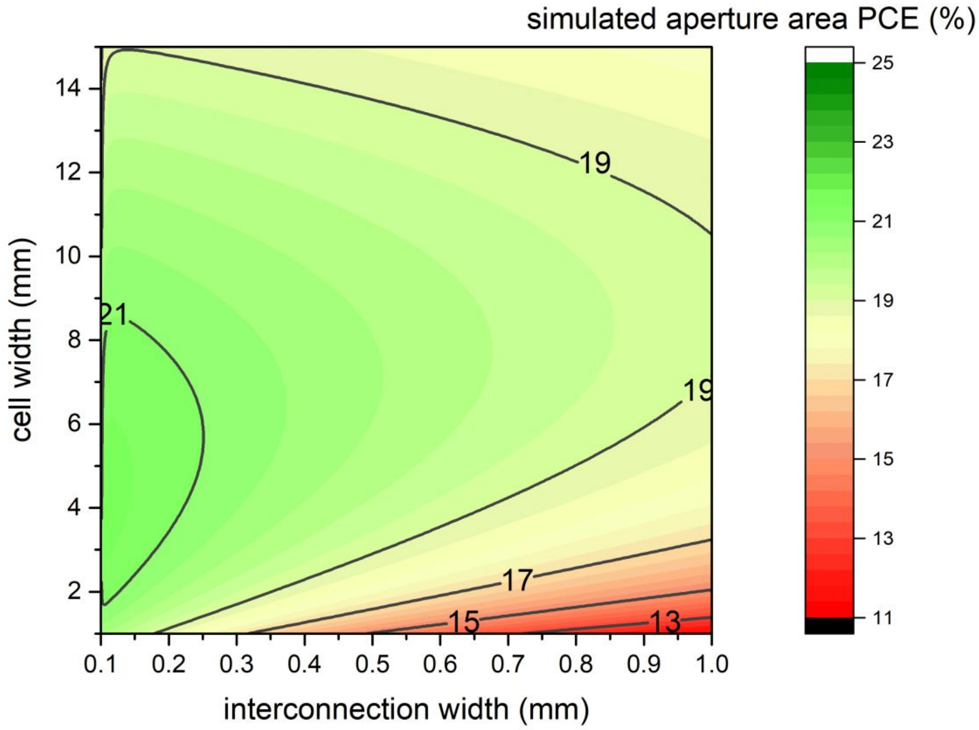

Figure S11. Simulated maximum efficiency (on aperture area) using the input of the 4-wire split measurement on 1 cm^2. The parameter used for the simulation are: conductivity of bottom electrode of 8 Ω sq^{-1}, transfer length of interconnection of 0.06 mm.

References

[1] Green, M. A. Accuracy of Analytical Expressions for Solar Cell Fill Factors. *Solar Cells,* **1982***, 7,* 337-340.

[2] Greulich, J.; Glatthaar, M.; Rein, S. Fill Factor Analysis of Solar Cells' Current–Voltage Curves. *Prog. Photovolt: Res. Appl*. **2010**, *18*, 511-515.
